# Patient and Caregiver Perceptions of Airway Clearance Methods Used for Cystic Fibrosis

**DOI:** 10.1155/2023/1422319

**Published:** 2023-07-28

**Authors:** Zoe E. Kienenberger, Tyler O. Farber, Mary E. Teresi, Francesca Milavetz, Sachinkumar B. Singh, Katie Larson Ode, Theodosia Thoma, Rebecca L. Weiner, Kathryn R. Burlage, Anthony J. Fischer

**Affiliations:** ^1^Pediatrics, University of Iowa, Iowa City, IA, USA; ^2^Department of Physical Therapy and Rehabilitation Science, University of Iowa, Iowa City, IA, USA

## Abstract

**Introduction:**

Cystic Fibrosis Foundation guidelines recommend people with CF perform daily airway clearance. This can be difficult for patients, as some find it time consuming or uncomfortable. Data comparing airway clearance methods are limited. We surveyed patients and their families to understand which methods are preferred and identify obstacles to performing airway clearance.

**Methods:**

We designed a REDCap survey and enrolled participants in 2021. Respondents reported information on airway clearance usage, time commitment, and medication use. They rated airway clearance methods for effectiveness, comfort, time commitment, importance, and compatibility with other treatments. The analysis included descriptive statistics and clustering.

**Results:**

60 respondents started and 52 completed the survey. The median patient age was 20 years. Respondents experienced a median of four airway clearance methods in their lifetime, including chest wall oscillation (vest, 92%), manual chest physical therapy (CPT, 88%), forced expiration technique (huff or cough, 77%), and exercise (75%). Past 30-day use was highest for exercise (62%) and vest (57%). The time commitment was generally less than 2 hours daily. Of those eligible for CFTR modulators, 53% reported decreased time commitment to airway clearance after starting treatment. On a scale of 0–100, respondents rated CFTR modulators as their most important treatment (median 99.5), followed by exercise (88). *Discussion*. Patients and caregivers are familiar with several methods of airway clearance for CF. They report distinct strengths and limitations of each method. Exercise and vest are the most common methods of airway clearance. The use of CFTR modulators may reduce patient-reported time commitment to airway clearance.

## 1. Introduction

Cystic fibrosis (CF) is an autosomal recessive disease caused by mutations in CFTR, a gene that encodes a chloride and bicarbonate channel [1]. Loss of CFTR function results in abnormal mucus in the respiratory tract [2, 3]. Because patients with CF have defects in mucociliary transport [2, 4], CF care guidelines recommend physical methods of airway clearance starting at an early age [5]. Several airway clearance methods are routinely used in CF, including a forced expiration technique (huff or cough), oscillating positive expiratory pressure devices (PEP), high-frequency chest wall oscillation (vest), and aerobic or resistance exercise training [6, 7]. While a randomized study comparing airway clearance techniques showed oscillating PEP devices protected patients with CF against exacerbations better than vest [8], other observational studies found no evidence that any specific airway clearance technique is superior to another [9, 10]. Some people with CF could favor certain forms of airway clearance depending on their age, developmental status, or disease progression. Differences between males and females in airway clearance practices were also reported [7].

While airway clearance treatments are almost universally prescribed, they can be challenging for patients and families across the age spectrum. Barriers to completing airway clearance may include time commitment, lack of financial resources, perceived lack of effectiveness, and discomfort. While care teams may recommend up to two hours per day of airway clearance, actual use of these methods is often far less than prescribed [11–13]. Completing airway clearance becomes increasingly difficult as children age [13] and are prescribed other therapies due to disease progression. The teenage years are a time of low adherence to airway clearance [11]. Teens manage their CF while maintaining busy academic, social, and extracurricular schedules with progressively less parental supervision. Historically, this correlates with a period of worsening lung function and increased hospitalizations [14]. In addition, socioeconomic factors significantly impact the ability to complete airway clearance. Families with maternal college education, annual income >$50,000, and more adults in the household have higher completion rates of airway clearance [11]. Airway clearance may be painful or unpleasant for some patients. In hospitalized patients at our center, we routinely observe refusal to complete physical therapy and other forms of airway clearance due to lack of energy, fatigue, shortness of breath, coughing, skin irritation, pain, or discomfort. Furthermore, the effectiveness of some methods of airway clearance has been questioned, particularly if not performed correctly [15].

The role of airway clearance for patients with CF could change with the introduction of CFTR modulator drugs [16–20]. These medications increase the activity of endogenous CFTR and cause immediate improvements in mucociliary transport [21], pulmonary air trapping [22], and lung function [16–20]. This raises questions about the future role or need for airway clearance in people with CF following an initiation of highly effective CFTR modulator drugs. Surveys of patients using CFTR modulators show that most (292/359, 81%) have not discontinued chronic treatments, although respondents ranked airway clearance techniques as their most burdensome treatment [23].

To assess the usage of airway clearance, identify barriers to regular use, and understand why some methods of airway clearance are preferred over others, we administered an anonymous survey to patients and caregivers in our pediatric and adult CF center. We hypothesized that adolescent patients would report less time using airway clearance methods compared to adults or younger children and that patients would prefer forms of airway clearance that are convenient or coordinate better with other therapies. In addition, we hypothesized that many forms of airway clearance are not completed because they are painful, unpleasant, or poorly tolerated. We considered that male and female patients could use different forms of airway clearance during adolescence and experience side effects differently. Finally, we predicted that patients taking CFTR modulators would report less airway clearance because of improved health.

## 2. Materials and Methods

### 2.1. Ethics Statement

This study was approved by the University of Iowa's Institutional Review Board (IRB), Study # 202008392. A waiver of documentation of consent was granted by the IRB. Subjects indicated their informed consent to participate in the study by their answer to the first question of the survey.

### 2.2. Survey Creation

We developed a survey in REDCap based in part on the Cystic Fibrosis Questionnaire-Revised (CFQ-R), a disease-specific instrument designed to measure the impact of CF on health, perceived well-being, and symptoms [24]. The survey was modified and expanded to obtain information on airway clearance usage, time commitment, and medications. The survey consisted of six sections including (1) demographics including age, gender, race, household size, education level, and current employment or educational status; (2) estimated time commitment for their treatments including airway clearance; (3) lifetime experience with airway clearance methods; (4) past 30-day airway clearance use; (5) current CF treatments including medications, CFTR modulator therapies, and airway clearance; and (6) perceived importance of each treatment currently in use by the patient.

Demographic information including age and sex was collected because we hypothesized that adolescent patients would be less likely to use airway clearance and that male and female patients might have different preferences. We analyzed patients in four different age categories: children aged 0–9 years, younger adolescents aged 10–14 years, older adolescents aged 15–18, and adults aged 19 and older. Information on socioeconomic status was requested, as this has been shown to correlate with airway clearance adherence in previous studies [11].

After the initial survey creation, several rounds of feedback were collected from CF center physicians, physical therapists, and pharmacists affiliated with the University of Iowa's Carver College of Medicine. A complete codebook for the survey is included in Supplemental Information.

### 2.3. Recruitment

Patients or parents/guardians of minor patients were approached during standard of care CF clinic visits at the University of Iowa Pediatric and/or Adult CF centers. All eligible patients or their caregivers were given an information sheet on how to access the computerized survey, which could be completed during the clinic visit or at home following the visit. The survey was voluntary and anonymous. Participants were enrolled over a 7-month period (January 2021to August 2021). The goal enrollment was 100 participants, which is roughly 40% of the combined size of the pediatric and adult cystic fibrosis centers at the University of Iowa. Surveys could be answered independently by a patient with CF or by a parent, guardian, partner, or spouse on their behalf. In this report, “respondent” refers to a person who answered the survey, and “patient” refers to a child or adult with CF whose health and airway clearance practices are the subject of the survey. The term “participant” is inclusive of both patients and respondents.

### 2.4. Statistical Analysis

RStudio (v1.2.5033) was used for statistical analysis. The analysis included descriptive statistics and clustering. Summary values reported in the text are median and interquartile range (IQR) unless indicated. Given the non-normal distribution of some variables, nonparametric tests including a Wilcoxon rank sum test (2 groups) or a Kruskal–Wallis test (3 or more groups) were performed. Fisher's exact test was used for proportions. Generalized linear modeling was utilized to analyze the relationship between airway clearance usage and perceived importance as a function of age, gender, and CFTR modulator usage. The heatmap function was used to display patient/caregiver opinions of the positive and negative attributes of each method of airway clearance.

## 3. Results

### 3.1. Summary of Survey Respondents

Surveys were provided to participants at regular CF clinic visits to the adult or pediatric CF centers at the University of Iowa. Demographic information is summarized in Table 1. Of the 174 surveys distributed in the clinic, 60 respondents started the survey and 52 completed the survey. Survey respondents identified themselves as either patients (*N* = 45) or parents/guardians (*N* = 14). 56 respondents identified the patient's gender, including 31 male and 25 female. The median age of the patients was 20 years (IQR 13.5–30.0). The self-reported race/ethnicity of the patients included White (51), American Indian/Alaska Native (1), Black/African American (1), and Other (2). The median FEV_1_ was 90% of what was predicted (IQR 76-110.5) among the 28 patients whose lung function results were recorded. Patient ages ranged from 0 to 75 years.

### 3.2. Familiarity with Airway Clearance Methods

Survey respondents reported familiarity with multiple forms of airway clearance (Table 2). The median lifetime number of airway clearance methods experienced per patient was 4 (IQR 3–5). High-frequency chest wall oscillation (vest), forced expiration techniques (huff or cough), manual chest physiotherapy, exercise, and oscillating PEP devices were the most reported methods.

### 3.3. Current Use of Airway Clearance Methods: Effect of Age and Gender

**Figure 1:**
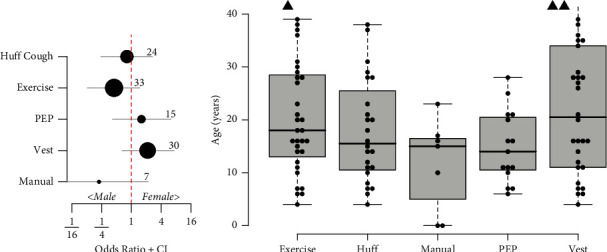
(a) Association of current (past 30-day) usage of airway clearance methods with gender. Filled circles indicate the odds ratio for females using the indicated methods for airway clearance. Gray lines are 95% confidence intervals; the lower confidence interval for manual CPT is truncated. The number of respondents reporting use of each method is indicated. (b) Current age of patients using different airway clearance methods. Subjects with age >40 are indicated by triangles. There were no significant differences in age between airway clearance methods, Kruskal–Wallis test *p*=0.17.

Past 30-day usage was highest for exercise and vest. Although 46 respondents reported experience with manual chest physical therapy (CPT) during their lifetimes, only 7 reported using manual CPT over the past 30 days, and none of the patients reported recent use of intrapulmonary percussive ventilation (IPV). There were trends towards higher current use of vest by females and exercise by males, but these were not statistically significant, as shown in Figure 1(a). Current use of manual CPT tended to be reported for younger patients, but there were no statistically significant differences (*p*=0.17) in age between current users of any of the common airway clearance methods, as shown in Figure 1(b).

### 3.4. Perceived Importance of Airway Clearance in Relationship to Other Therapies

**Figure 2:**
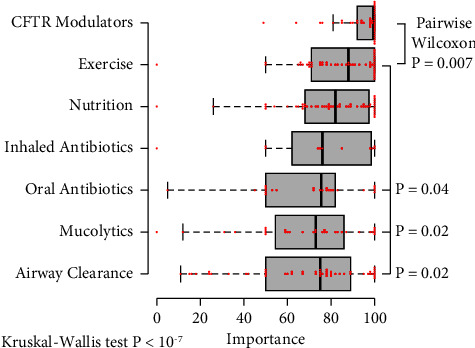
Perceived importance of different therapies for cystic fibrosis as rated by patients or caregivers. Users scored a visual analog scale to rate the importance of different therapies. The default position of the slider was 50 on a scale of 0–100. Data points indicate individual responses; boxplots indicate median and quartiles. CFTR modulators were ranked as significantly more important than all other treatments. Exercise was rated more important than oral antibiotics, mucolytics, and other forms of airway clearance.

People with CF receive many classes of treatment in addition to airway clearance, including nutritional therapies, antibiotics, mucolytic drugs, and CFTR modulators. Survey respondents were asked which classes of treatment they use. For each treatment class, respondents were asked to rate the importance of that treatment to their health on a visual analog sliding scale. The scale records values from 0 to 100, with the default slider position set to 50. Respondents regarded CFTR modulator therapies as the most important treatment class, with a median importance of 99.5 (IQR 92.5–100, *p* < 0.0001) (Figure 2). Exercise was considered less important than CFTR modulators (median importance 88, IQR 71–100, *p*=0.007 vs. CFTR modulators), but more important than other forms of airway clearance, mucolytics, or oral antibiotics (all *p* < 0.05 vs. exercise). There were no significant differences between the remaining classes of treatments. For each of these remaining treatment classes, including airway clearance, the median importance was rated significantly higher than the default value of 50.

### 3.5. Importance of Airway Clearance: Role of Sex and CFTR Modulator Therapy

There was a wide dispersion in the perceived importance of airway clearance (Figure 2). To understand which factors potentially influence patient attitudes about airway clearance, a multivariable regression analysis was performed to determine whether age, sex, or CFTR modulator use were associated with the perceived importance of airway clearance (Supplemental Data 1). Respondents rated airway clearance lower in importance if the patient was male (effect size 18 points lower, *p* = 0.02). Age was not statistically significant in this model.

Exercise is regarded by some as an alternative form of airway clearance [25, 26]. In this survey, exercise was considered important for maintaining health by most respondents. In contrast to other methods of airway clearance, the perceived importance of exercise was rated highly regardless of age, sex, or CFTR modulator use (see Supplemental Data 2). Because patients and their parents may have different opinions on the importance of treatments for CF therapies, we compared responses between patients who completed the survey themselves and proxy responses given by parents. We found that the responses of patients and parents were similar for each type of treatment (Supplemental Data 3).

### 3.6. Reported Time Commitment for Airway Clearance

**Figure 3:**
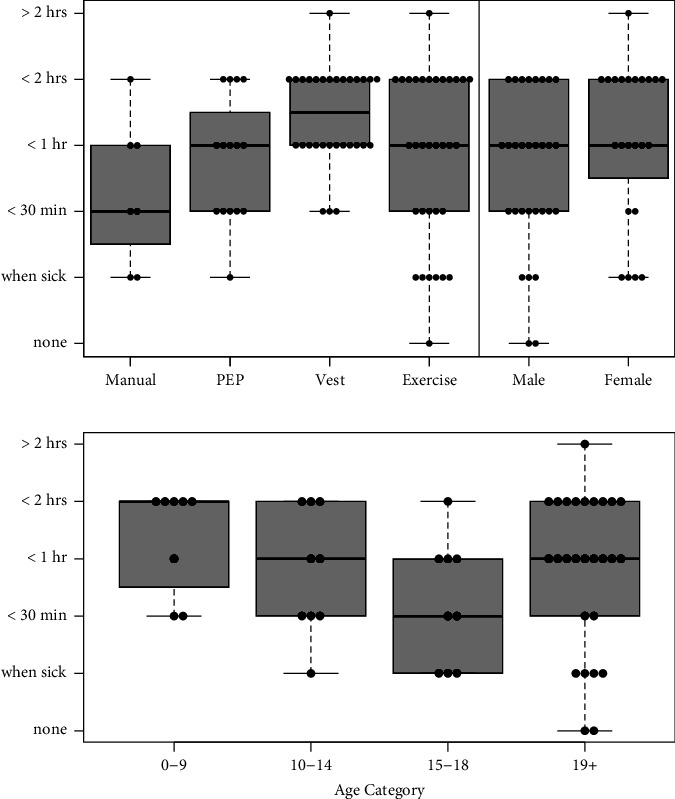
(a) Time commitment to CF treatments versus airway clearance methods and gender, *N* = 54 responses. Data points indicate total time estimated for treatments among respondents who used the indicated airway clearance methods at bottom. Some respondents reported current use of multiple methods. Vest users reported more time commitment to daily treatments. In a generalized linear model of time commitment by age category, gender, CFTR modulator use, and airway clearance method, vest usage was independently associated with the most reported time for treatments (*p*=0.0001). (b) Reported time commitment by age category among survey respondents. Age categories were defined as child (0–9 years), young adolescents (10–14 years), older adolescents (15–18 years), and adults >19 years. There was a trend toward lower median time commitment in older adolescent patients.

Airway clearance treatments can be time-consuming. Survey respondents were asked to select the amount of time spent on daily treatments from a list of choices including none, only when sick, less than 30 minutes, 30–59 minutes, 1-2 hours, or more than 2 hours. The total daily time required to complete all treatments was compared to the past 30-day use of different airway clearance methods. The most common response provided was between 1 and 2 hours per day on treatments. However, 19% of respondents reported spending less than 30 minutes per day (Supplemental Data 3). Respondents who used vest therapies for airway clearance within the past 30 days had the highest median time commitment (Figure 3). There was a trend towards more time commitment to airway clearance for female patients, although this was not statistically significant. Time commitment did not correlate significantly with age, CFTR modulator use, or any type of airway clearance method other than vest (*p*=0.0001 for vest, *p* > 0.05 for all other associations). Over half of respondents receiving a CFTR modulator (23/43 = 53%) reported that treatment time for airway clearance had been reduced since starting this medication (Table 3).

### 3.7. Positive Attributes of Different Airway Clearance Methods

**Figure 4:**
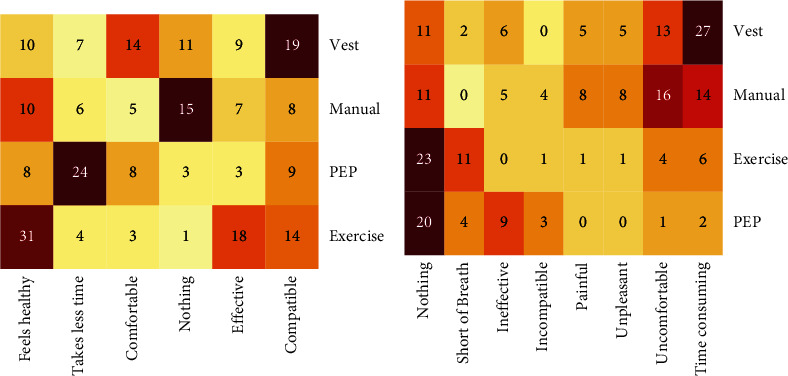
Heatmap display of patient/caregiver opinions of airway clearance methods. Users could rate positive attributes (a) or negative attributes (b) of any airway clearance method they had experienced. Colored are scaled by rows, with common responses indicated by darker colors. The number of responses is given in each cell.

To determine why some airway clearance methods are preferred, survey respondents were asked to indicate what they like about each airway clearance method they have used in their lifetime. Respondents could make multiple selections, including whether the method takes little time, is comfortable, works well with other treatments, is effective, or feels good or healthy. To allow for neutral or negative responses to the question, choices of “nothing,” “other,” and “I don't know” were provided. The positive attributes for each airway clearance method were tabulated, and clustering analysis was performed to determine which of these attributes was associated with each method (Figure 4(a)). Vest users rated this method as being compatible with other treatments. Exercise was the most likely to be selected as healthy or feeling good. Users liked oscillating PEP devices because of low time commitment.

### 3.8. Negative Attributes of Different Airway Clearance Methods

Survey respondents were asked to indicate what they disliked about each method of airway clearance they had experienced in their lifetime. Respondents could select multiple options, including shortness of breath, time consuming, painful, uncomfortable, less effective than other treatments, not compatible with other treatments, or if the method is unpleasant or embarrassing. To allow for neutral/positive responses to the question for each method, choices of “nothing” and “I don't know” were provided. The most common reason people disliked vest treatment was time commitment (Figure 4(b)). Respondents reported that manual CPT was uncomfortable, exercise was most likely to cause shortness of breath, and oscillating PEP devices were most likely to be considered ineffective.

### 3.9. Location of Pain or Discomfort Caused by Different Airway Clearance Methods

Manual CPT and vest users were the most likely to report pain and discomfort. The most common locations for pain or discomfort were the chest (*N* = 19), abdomen (*N* = 12), back (*N* = 10), and breasts (*N* = 10) (Table 4).

## 4. Discussion

In this population, patients with CF or their caregivers were familiar with several forms of airway clearance and reported ∼1 hour of daily time commitment to these therapies. Those receiving CFTR modulators reported decreasing their airway clearance, and some respondents reported performing little (<30 minutes per day) airway clearance.

While respondents were familiar with many forms of airway clearance, their past 30-day usage indicated preference for exercise, followed by vest, forced exhalation (huff or cough), and oscillating PEP devices. Male and female patients used each of the airway clearance forms roughly equally, but there were trends towards greater vest usage in females and exercise in males, which is consistent with observations in prior studies [26, 27]. Although we hypothesized there would be differences in airway clearance practices by age group, children, adolescents, and adults reported similar use of airway clearance methods and overall time commitment. The lack of significant differences between age categories in time spent on airway clearance could be related to the relatively small sample size in this study. The most important contributor to time spent on airway clearance in our cohort was the use of a vest device.

Whether exercise is an acceptable substitute for traditional airway clearance remains controversial [6, 25, 28, 29]. Exercise is common in patients with CF, and many patients either incorporate exercise into their airway clearance or use exercise as a replacement for traditional airway clearance [25]. Exercise may improve the ease of expectoration and sputum clearance and may have similar effects to traditional airway clearance in the short term [30]. Some advocate the use of exercise as airway clearance in part because exercise is more easily accepted by society, which helps to normalize the lives of patients with CF [29]. However, others contend that traditional airway clearance techniques are still the superior method for mucus clearance [28].

Despite the inherent challenges of exercise and airway clearance, many respondents considered these therapies important for maintaining their health. However, it remains unclear which forms of airway clearance are optimal for patients with CF. A randomized comparison of an oscillating PEP device versus high-frequency chest wall oscillation in patients 6 or older showed fewer exacerbations in patients randomized to the oscillating PEP device, but no significant differences in lung function [8]. These results should be interpreted with caution in children younger than 6, children with respiratory muscle weakness, or patients unable to follow directions. Many authors suggest no method of airway clearance is superior to others [30–32]. Even within a class of airway clearance treatments, there is significant variability. For instance, there is no clear evidence that one type of oscillating PEP device is superior to another [31]. Our goal was not to determine which method of airway clearance was best, but rather to identify barriers to using airway clearance methods. Patients with CF or their caregivers may wish to use airway clearance techniques that best meet their individual needs after considering comfort, convenience, flexibility, practicality, and cost [32].

### 4.1. Advantages

An advantage of this study is that our survey compares airway clearance methods from the patient's perspective. This could provide a better understanding of why some methods are preferred by patients. Knowing how patients think or feel about airway clearance methods could help clinicians understand potential barriers to using airway clearance, including pain or discomfort, time commitment, and perceived lack of efficacy. This study also compares the importance of airway clearance relative to medications and other CF therapies from the perspective of the patient. This information may help the CF research community plan future studies aimed at simplifying CF care plans.

### 4.2. Limitations

Because this study was voluntary and the response rate to survey requests was low, there is some risk of selection or responder bias. The study was anonymous and used self-reported time commitments. Thus, we could not confirm the time spent on airway clearance by an independent objective measure. Our questionnaire used a visual analog scale to allow respondents to rate the importance of different treatments on a wide scale of 0–100 rather than a limited number of choices. This established that patients consider CFTR modulators very important but given that the scale has a default value of 50, it leaves some uncertainty on how important patients consider other treatments. Finally, this was a single-center study, which potentially limits generalizability to other centers that care for patient populations with greater ethnic diversity or have lower eligibility for CFTR modulator treatments. However, the distributions of age, gender, CFTR theratypes, and pulmonary function among survey responses were similar to our recent reports [33, 34]. Thus, the results of this survey are likely to be representative for our center.

## 5. Conclusion

Patients with CF are familiar with several methods of airway clearance. In this study, people with CF or their caregivers consider CFTR modulators more important than airway clearance, and respondents receiving these drugs report decreased time spent on airway clearance. In addition, our survey indicates people with CF and their caregivers prefer some methods of airway clearance over others and may consider exercise as an adequate replacement for traditional forms of airway clearance. Despite this, people with CF still consider airway clearance important for maintaining their health.

## Figures and Tables

**Table 1:** Demographic characteristics.

Characteristics	Numbers	Percent^*∗*^
*Identity of respondent*	59	
Patient	45	76%
(i) 18 years or older	31	53%
(ii) 17 years or younger	14	24%
Parent/guardian	14	24%
Spouse	0	0%

*Patient's gender*	56	
Male	31	55%
Female	25	45%
Nonbinary	0	0%
Transgender female	0	0%
Transgender male	0	0%
Others	0	0%
Prefer not to say	0	0%

*Patient's race/ethnicity* ^†^	52	
White	51	98%
Others	2	4%
American Indian/Alaska native	1	2%
Black/African American	1	2%
Asian	0	0%
Hispanic/Latinx	0	0%
Native Hawaiian/Pacific Islander	0	0%

*CFTR modulator*	53	
Ivacaftor	2	4%
Lumacaftor/ivacaftor	1	2%
Tezacaftor/ivacaftor	2	4%
Elexacaftor/tezacaftor/ivacaftor	38	72%
None of these	10	19%

	Median	IQR

Patient's age, *N* = 56	20	[13.5 – 30.0]
^‡^FEV_1_% predicted, *N* = 28	90	[76.0 – 110.5]

The number of completed responses for each question is given. ^*∗*^Percentage is calculated based on the number of complete responses for each question. ^†^Respondents could choose more than one entry; percentages may exceed 100%. ^‡^Patient reported spirometry results. *N* = 4 responses were removed because raw FEV_1_ values were reported rather than FEV_1_% predicted.

**Table 2:** Reported lifetime experience and the current use of airway clearance methods.

Methods^*∗*^	Lifetime use, *N* = 52 responses					Past 30-day use, *N* = 53 responses				
	Male	Female	NR^†^	Total	(%)	Male	Female	NR^†^	Total	(%)
Exercise	21	17	1	39	75	21	12	0	33	62
Vest	25	22	1	48	92	14	16	0	30	57
Huff or cough	22	17	1	40	77	14	10	0	24	45
Oscillating PEP	18	16	1	35	67	7	8	0	15	28
Manual CPT	22	23	1	46	88	5	1	1	7	13
Others	5	2	0	7	13	2	0	0	2	4
IPV	2	4	0	6	12	0	0	0	0	0
None	0	0	0	0	0	1	0	0	1	2

^*∗*^Respondents could choose more than one method of airway clearance. ^†^NR = no response was provided for gender. Methods are listed in order by frequency of past 30-day use.

**Table 3:** Change in time commitment to airway clearance after starting the CFTR modulator therapy.

CFTR modulators	Time commitment			Total
	More	Same	Less	
Ivacaftor	0	1	1	2
Lumacafor/ivacaftor or Tezacaftor/ivacaftor	0	2	1	3
Elexacaftor/tezacaftor/ivacaftor	1	16	21	38
Total	1	19	23	43

**Table 4:** Location of pain or discomfort experienced by patients using different methods of airway clearance.

Sites	Manual	Vest	Oscillating PEP	Exercise	Any method
Chest	12	12	1	4	19
Abdomen	4	7	0	2	12
Back	6	4	0	3	10
Breasts	7	4	0	0	10
Armpits	3	5	0	0	7
Headache	1	1	0	3	4
Neck	2	1	0	0	3
Port or PICC^*∗*^	1	2	0	0	3
Arm or leg	0	1	0	2	3
Throat	0	0	0	1	1
Others	3	1	0	0	4

^*∗*^Peripherally inserted central catheter.

## Data Availability

The survey instrument may be used with written permission from the authors. Anonymous survey responses are available to qualified investigators after completion of a materials transfer agreement.

## Abbreviations

CPT: Chest physical therapy
IPV: Intrapulmonary percussive ventilation
PEP: Oscillating positive expiratory pressure device
Vest: High-frequency chest wall oscillation.

## Supplementary Materials

Supplementary Materials Supplemental Data 1. Perceived importance of airway clearance importance by age and gender. Supplemental Data 2. Perceived importance of exercise importance by age and gender. Supplemental Data 3. Perceived importance of airway clearance, stratified by survey respondent (self vs. proxy respondent). REDCap Codebook.
