# Supplementary material for: Patient and Caregiver Perceptions of Airway Clearance Methods Used for Cystic Fibrosis
Source: Can Respir J. 2023 Jul 28;2023:1422319. doi: 10.1155/2023/1422319 (PMC10403321; doi:10.1155/2023/1422319)
Supplement: Supplementary Materials — Supplemental Data 1. Perceived importance of airway clearance importance by age and gender. Supplemental Data 2. Perceived importance of exercise importance by age and gender. Supplemental Data 3. Perceived importance of airway clearance, stratified by survey respondent (self vs. proxy respondent). REDCap Codebook. [file 1422319.f1.zip › Airway Clearance Manuscript Supplement 12_5_2022.docx]

**Supplemental Data**

**Patient and caregiver perceptions of
airway clearance methods used for cystic fibrosis**

by

Zoe E. Kienenberger, Tyler O. Farber, Mary E. Teresi, Francesca Milavetz,
Sachinkumar B. Singh, Katie Larson Ode, Theodosia Thoma, Rebecca L. Weiner,
Kathryn R. Burlage, and Anthony J. Fischer

**Supplemental Data 1**. Perceived importance of airway clearance importance by age and gender. Female respondents are indicated with red symbols. In a generalized linear model, including age, gender, and CFTR modulator use as factors, male gender (P = 0.023, -18 units) was associated with less perceived importance of airway clearance.

**Supplemental Data 2**. Perceived importance of exercise importance by age and gender, female patients are indicated with red symbols. There was no significant association of the perceived importance of exercise with gender, age, or CFTR modulator use.

**Supplemental Data 3**. Perceived importance of airway clearance, stratified by survey respondent.

|  | **Mean Importance** | |  |
| --- | --- | --- | --- |
|  | **Self Respondent** | **Proxy Respondent** |  |
| **Treatment** | **N = 45** | **N = 14** | ***P**** |
| Airway Clearance | 66.2 | 76.9 | 0.15 |
| Mucolytics | 66.7 | 73.7 | 0.43 |
| Oral Antibiotics | 67.8 | 74.9 | 0.42 |
| Inhaled Antibiotics | 73.4 | -^†^ | - |
| Nutrition | 77.7 | 85.5 | 0.16 |
| Exercise | 83.3 | 84.0 | 0.89 |
| CFTR modulator | 94.2 | 93.0 | 0.74 |

*Welch’s t-test. ^†^No proxy respondents provided a rating for inhaled antibiotics.
