# Supplementary material for: Patient and Caregiver Perceptions of Airway Clearance Methods Used for Cystic Fibrosis
Source: Can Respir J. 2023 Jul 28;2023:1422319. doi: 10.1155/2023/1422319 (PMC10403321; doi:10.1155/2023/1422319)
Supplement: Supplementary Materials — Supplemental Data 1. Perceived importance of airway clearance importance by age and gender. Supplemental Data 2. Perceived importance of exercise importance by age and gender. Supplemental Data 3. Perceived importance of airway clearance, stratified by survey respondent (self vs. proxy respondent). REDCap Codebook. [file 1422319.f1.zip › REDCap Codebook.pdf]

Data Dictionary Codebook

05/05/2022 2:27pm

	#	Variable / Field Name	Field Label<br>*Field Note*	Field Attributes (Field Type, Validation, Choices, Calculations, etc.)
Instrument: **PT Survey** (pt_survey) Enabled as survey				
	1	[record_id]	Record ID	text
	2	[consent]	Section Header: *[If you are an adult or a minor child who will complete the survey, 'you' means 'you'. If you are a parent/guardian completing the survey for your child, 'you' means 'your child'] We are inviting you to participate in a research study. The purpose of the study is to determine which types of airway clearance you use and why you may or may not use them routinely. We are inviting you to be in this study because you have cystic fibrosis. Approximately 200 people will take part in this study at the University of Iowa. If you agree to participate, we would like you to complete the following survey, which will take about 10 minutes. If you do not want to participate, you may exit the survey. You are free to skip any questions you would prefer not to answer. The information you provide may be used in separate future research studies. We will keep the information you provide confidential, however federal regulatory agencies and the University of Iowa Institutional Review Board (a committee that reviews and approves research studies) may inspect and copy records pertaining to this research. None of the information we collect will be able to identify you individually and we will not maintain a link between you and your data. This data can be used for future research, but again, you will not be able to be individually identified. If we write a report about this study we will do so in such a way that you cannot be identified. There are no known risks from being in this study, and you will not benefit personally. However, we hope that others may benefit in the future from what we learn as a result of this study. You will not have any costs for being in this research study. You will not be paid for being in this research study. Taking part in this research study is completely voluntary. If you decide not to be in this study, or if you stop participating at any time, you won't be penalized or lose any benefits for which you otherwise qualify. If you have any questions about the research study itself, please contact Anthony Fischer at 319-356-6992. If you experience a research-related injury, please contact: Anthony Fischer at 319-356-6992. If you have questions about the rights of research subjects, please contact the Human Subjects Office, 105 Hardin Library for the Health Sciences, 600 Newton Rd, The University of Iowa, Iowa City, IA 52242-1098, (319) 335-6564, or e-mail irb@uiowa.edu. To offer input about your experiences as a research subject or to speak to someone other than the research staff, call the Human Subjects Office at the number above. Thank you very much for your consideration. Sincerely, Anthony Fischer, MD*<br>Do you agree to participate in this survey? You may withdraw your participation at any time during the survey.	yesno<br>1 Yes<br>0 No<br>Stop actions on 0
	3	[who_filled_out]	Section Header: *Who is filling out this survey?*<br>Are you the patient or the caregiver (parent / legal guardian)?	radio<br>1 Patient (17 years or younger)<br>2 Patient (18 years or older)<br>3 Parent / Legal Guardian
	4	[year_of_birth]	Section Header: *Demographics*<br>Patient's Year of Birth	text (integer, Min: 1920, Max: 2020), Identifier
	5	[patient_current_age]	Patient's age in years (whole number)<br>example: if you are 12.5, enter 12	text (integer, Min: 0, Max: 100), Identifier<br>Custom alignment: RH
	6	[gender]	Patient's Gender	radio, Identifier<br>1 Male<br>2 Female<br>3 Non-binary<br>4 Transgender female<br>5 Transgender male<br>6 Other<br>7 Prefer not to say

	7	[chest_binder]<br><br>Show the field ONLY if:<br>[gender] = '5'	Do you (THE PATIENT) currently use a chest binder?	yesno<br>1 Yes<br>0 No
	8	[disclaimer1]	For the duration of the survey, "you" or "your" or "I" refers to the patient	descriptive
	9	[time_on_treatments]	Section Header: *Over the last two weeks:*<br><br>How much time do you spend each day on your treatments?	radio<br>1 Less than 30 minutes<br>2 30 minutes to 59 minutes<br>3 1 to 2 hours<br>4 More than 2 hours<br>5 I don't take my treatments<br>6 I don't take my treatments unless I'm sick<br>7 I don't know
	10	[doing_treatments]	How difficult is it for you to do your treatments (including medications and airway clearance) each day?	radio<br>1 Not at all<br>2 A little<br>3 Moderately<br>4 Very
	11	[treatments_daily_life]	How much do your treatments make your daily life more difficult?	radio<br>1 Not at all<br>2 A little<br>3 Moderately<br>4 A lot
	12	[overall_health]	How is your overall health?	radio<br>1 Excellent<br>2 Good<br>3 Fair<br>4 Poor
	13	[fev_known]	Do you know your percent predicted forced expiratory volume (ppFEV1)?	yesno<br>1 Yes<br>0 No
	14	[fev]<br><br>Show the field ONLY if:<br>[fev_known] = '1'	What is your percent predicted forced expiratory volume (ppFEV1)?	text (number)
	15	[ever_used]	Section Header: *Airway Clearance*<br><br>In your life, which forms of airway clearance have you EVER USED?<br>Select ALL that apply	checkbox<br>1 ever_used___1 Manual Percussion (Chest PT)<br>2 ever_used___2 Intrapulmonary Percussive Ventilation (IPV)<br>3 ever_used___3 High Frequency Chest Wall Oscillation (Vest)<br>4 ever_used___4 Oscillating Positive Expiratory Pressure (PEP), e.g. Aerobika or Acapella<br>5 ever_used___5 Vigorous Exercise e.g. running<br>6 ever_used___6 Huff Coughing<br>7 ever_used___7 Other<br>8 ever_used___8 I don't know

<table>
<tr><td></td><td>16</td><td>[clearance_forms]</td><td>Which forms of airway clearance have you used in the PAST 30 DAYS?
Select ALL that apply</td><td>checkbox
<table>
<tr><td>1</td><td>clearance_forms___1</td><td>Manual Percussion (Chest PT)</td></tr>
<tr><td>2</td><td>clearance_forms___2</td><td>Intrapulmonary Percussive Ventilation (IPV)</td></tr>
<tr><td>3</td><td>clearance_forms___3</td><td>High Frequency Chest Wall Oscillation (Vest)</td></tr>
<tr><td>4</td><td>clearance_forms___4</td><td>Oscillating Positive Expiratory Pressure (PEP), e.g. Aerobika or Acapella</td></tr>
<tr><td>5</td><td>clearance_forms___5</td><td>Vigorous Exercise e.g. running</td></tr>
<tr><td>6</td><td>clearance_forms___6</td><td>Huff Coughing</td></tr>
<tr><td>7</td><td>clearance_forms___7</td><td>None</td></tr>
<tr><td>8</td><td>clearance_forms___8</td><td>Other</td></tr>
<tr><td>9</td><td>clearance_forms___9</td><td>I don't know</td></tr>
</table></td></tr>
<tr><td></td><td>17</td><td>[why_no_like]

Show the field ONLY if:
[clearance_forms(7)] = '1'</td><td>Why don't you perform any of these airway clearance techniques?
Select ALL that apply</td><td>checkbox
<table>
<tr><td>1</td><td>why_no_like___1</td><td>I get short of breath</td></tr>
<tr><td>2</td><td>why_no_like___2</td><td>They take too much time</td></tr>
<tr><td>3</td><td>why_no_like___3</td><td>They are painful</td></tr>
<tr><td>4</td><td>why_no_like___4</td><td>They are uncomfortable</td></tr>
<tr><td>5</td><td>why_no_like___5</td><td>They are less effective than medicine</td></tr>
<tr><td>6</td><td>why_no_like___6</td><td>They do not coordinate easily with other treatments</td></tr>
<tr><td>7</td><td>why_no_like___7</td><td>They are unpleasant or embarrassing</td></tr>
<tr><td>8</td><td>why_no_like___8</td><td>I do not like them</td></tr>
<tr><td>9</td><td>why_no_like___9</td><td>Other</td></tr>
<tr><td>10</td><td>why_no_like___10</td><td>I don't know</td></tr>
</table></td></tr>
<tr><td></td><td>18</td><td>[chest_pt_like]

Show the field ONLY if:
[ever_used(1)] = '1'</td><td>What do you LIKE about Manual Percussion (Chest PT)?
Select ALL that apply</td><td>checkbox
<table>
<tr><td>1</td><td>chest_pt_like___1</td><td>It takes little time</td></tr>
<tr><td>2</td><td>chest_pt_like___2</td><td>It is comfortable</td></tr>
<tr><td>3</td><td>chest_pt_like___3</td><td>It works well with my other treatments</td></tr>
<tr><td>4</td><td>chest_pt_like___4</td><td>It is more effective than other forms of airway clearance</td></tr>
<tr><td>5</td><td>chest_pt_like___5</td><td>It feels good or is healthy</td></tr>
<tr><td>6</td><td>chest_pt_like___6</td><td>Nothing</td></tr>
<tr><td>7</td><td>chest_pt_like___7</td><td>Other</td></tr>
<tr><td>8</td><td>chest_pt_like___8</td><td>I don't know</td></tr>
</table></td></tr>
<tr><td></td><td>19</td><td>[other_like_chest_pt]

Show the field ONLY if:
[chest_pt_like(7)] = '1'</td><td>Please explain what you LIKE about Manual Percussion (Chest PT)</td><td>notes</td></tr>
</table>

	20	[ipv_like]<br><br>Show the field ONLY if:<br>[ever_used(2)] = '1'	What do you LIKE about Intrapulmonary Percussive Ventilation (IPV)?<br>Select ALL that apply	checkbox<br>1 ipv_like___1 It takes little time<br>2 ipv_like___2 It is comfortable<br>3 ipv_like___3 It works well with my other treatments<br>4 ipv_like___4 It is more effective than other forms of airway clearance<br>5 ipv_like___5 It feels good or is healthy<br>6 ipv_like___6 Nothing<br>7 ipv_like___7 Other<br>8 ipv_like___8 I don't know
	21	[other_like_ipv]<br><br>Show the field ONLY if:<br>[ipv_like(7)] = '1'	Please explain what you LIKE about Intrapulmonary Percussive Ventilation (IPV)	notes
	22	[vest_like]<br><br>Show the field ONLY if:<br>[ever_used(3)] = '1'	What do you LIKE about High Frequency Chest Wall Oscillation (Vest)?<br>Select ALL that apply	checkbox<br>1 vest_like___1 It takes little time<br>2 vest_like___2 It is comfortable<br>3 vest_like___3 It works well with my other treatments<br>4 vest_like___4 It is more effective than other forms of airway clearance<br>5 vest_like___5 It feels good or is healthy<br>6 vest_like___6 Nothing<br>7 vest_like___7 Other<br>8 vest_like___8 I don't know
	23	[other_like_vest]<br><br>Show the field ONLY if:<br>[vest_like(7)] = '1'	Please explain what you LIKE about High Frequency Chest Wall Oscillation (Vest)	notes
	24	[pep_like]<br><br>Show the field ONLY if:<br>[ever_used(4)] = '1'	What do you LIKE about Oscillating Positive Expiratory Pressure (PEP), e.g. Aerobika or Acapella?<br>Select ALL that apply	checkbox<br>1 pep_like___1 It takes little time<br>2 pep_like___2 It is comfortable<br>3 pep_like___3 It works well with my other treatments<br>4 pep_like___4 It is more effective than other forms of airway clearance<br>5 pep_like___5 It feels good or is healthy<br>6 pep_like___6 Nothing<br>7 pep_like___7 Other<br>8 pep_like___8 I don't know
	25	[other_like_pep]<br><br>Show the field ONLY if:<br>[pep_like(7)] = '1'	Please explain what you LIKE about Oscillating Positive Expiratory Pressure (PEP), e.g. Aerobika or Acapella	notes

	26	[exercise_like]<br><br>Show the field ONLY if:<br>[ever_used(5)] = '1'	What do you LIKE about Vigorous Aerobic Exercise?<br>Select ALL that apply	checkbox<br>1 exercise_like___1 It takes little time<br>2 exercise_like___2 It is comfortable<br>3 exercise_like___3 It works well with my other treatments<br>4 exercise_like___4 It is more effective than other forms of airway clearance<br>5 exercise_like___5 It feels good or is healthy<br>6 exercise_like___6 Nothing<br>7 exercise_like___7 Other<br>8 exercise_like___8 I don't know
	27	[other_like_exercise]<br><br>Show the field ONLY if:<br>[exercise_like(7)] = '1'	Please explain what you LIKE about Vigorous Aerobic Exercise	notes
	28	[huff_cough_like]<br><br>Show the field ONLY if:<br>[ever_used(6)] = '1'	What do you LIKE about Huff Coughing?<br>Select ALL that apply	checkbox<br>1 huff_cough_like___1 It takes little time<br>2 huff_cough_like___2 It is comfortable<br>3 huff_cough_like___3 It works well with my other treatments<br>4 huff_cough_like___4 It is more effective than other forms of airway clearance<br>5 huff_cough_like___5 It feels good or is healthy<br>6 huff_cough_like___6 Nothing<br>7 huff_cough_like___7 Other<br>8 huff_cough_like___8 I don't know
	29	[other_like_huff_cough]<br><br>Show the field ONLY if:<br>[huff_cough_like(7)] = '1'	Please explain what you LIKE about Huff Coughing	notes
	30	[chest_pt_dislike]<br><br>Show the field ONLY if:<br>[ever_used(1)] = '1'	What do you DISLIKE about Manual Percussion (Chest PT)?<br>Select ALL that apply	checkbox<br>1 chest_pt_dislike___1 I get short of breath<br>2 chest_pt_dislike___2 It takes too long<br>3 chest_pt_dislike___3 It is painful<br>4 chest_pt_dislike___4 It is uncomfortable<br>5 chest_pt_dislike___5 It is less effective than other forms of airway clearance<br>6 chest_pt_dislike___6 It does not work well with my other treatments<br>7 chest_pt_dislike___7 It is unpleasant or embarrassing<br>8 chest_pt_dislike___8 Nothing<br>9 chest_pt_dislike___9 I don't know
	31	[chest_pt_follow_up]<br><br>Show the field ONLY if:<br>[chest_pt_dislike(3)] = '1' or [chest_pt_dislike(4)] = '1' or [chest_pt_dislike(7)] = '1'	Why is Manual Percussion (Chest PT) painful, uncomfortable or unpleasant?	text

<table>
<tr><td></td><td>32</td><td>[chest_pt_where]

Show the field ONLY if:
[chest_pt_dislike(3)] = '1' or [chest_pt_dislike(4)] = '1' or [chest_pt_dislike(7)] = '1'</td><td>Where on your body does Manual Percussion (Chest PT) bother you?
Select ALL that apply</td><td>checkbox
<table>
<tr><td>1</td><td>chest_pt_where___1</td><td>head</td></tr>
<tr><td>2</td><td>chest_pt_where___2</td><td>mouth</td></tr>
<tr><td>3</td><td>chest_pt_where___3</td><td>throat</td></tr>
<tr><td>4</td><td>chest_pt_where___4</td><td>neck</td></tr>
<tr><td>5</td><td>chest_pt_where___5</td><td>back</td></tr>
<tr><td>6</td><td>chest_pt_where___6</td><td>chest</td></tr>
<tr><td>7</td><td>chest_pt_where___7</td><td>armpits</td></tr>
<tr><td>8</td><td>chest_pt_where___8</td><td>breasts</td></tr>
<tr><td>9</td><td>chest_pt_where___9</td><td>stomach/abdomen</td></tr>
<tr><td>10</td><td>chest_pt_where___10</td><td>my port or PICC line</td></tr>
<tr><td>11</td><td>chest_pt_where___11</td><td>my arms or legs</td></tr>
<tr><td>12</td><td>chest_pt_where___12</td><td>other</td></tr>
</table></td></tr>
<tr><td></td><td>33</td><td>[ipv_dislike]

Show the field ONLY if:
[ever_used(2)] = '1'</td><td>What do you DISLIKE about Intrapulmonary Percussive Ventilation (IPV)?
Select ALL that apply</td><td>checkbox
<table>
<tr><td>1</td><td>ipv_dislike___1</td><td>I get short of breath</td></tr>
<tr><td>2</td><td>ipv_dislike___2</td><td>It takes too long</td></tr>
<tr><td>3</td><td>ipv_dislike___3</td><td>It is painful</td></tr>
<tr><td>4</td><td>ipv_dislike___4</td><td>It is uncomfortable</td></tr>
<tr><td>5</td><td>ipv_dislike___5</td><td>It is less effective than other forms of airway clearance</td></tr>
<tr><td>6</td><td>ipv_dislike___6</td><td>It does not work well with my other treatments</td></tr>
<tr><td>7</td><td>ipv_dislike___7</td><td>It is unpleasant or embarrassing</td></tr>
<tr><td>8</td><td>ipv_dislike___8</td><td>Nothing</td></tr>
<tr><td>9</td><td>ipv_dislike___9</td><td>I don't know</td></tr>
</table></td></tr>
<tr><td></td><td>34</td><td>[ipv_follow_up]

Show the field ONLY if:
[ipv_dislike(3)] = '1' or [ipv_dislike(4)] = '1' or [ipv_dislike(7)] = '1'</td><td>Why is Intrapulmonary Percussive Ventilation (IPV) painful, uncomfortable or unpleasant?</td><td>text</td></tr>
<tr><td></td><td>35</td><td>[ipv_where]

Show the field ONLY if:
[ipv_dislike(3)] = '1' or [ipv_dislike(4)] = '1' or [ipv_dislike(7)] = '1'</td><td>Where on your body does Intrapulmonary Percussive Ventilation (IPV) bother you?
Select ALL that apply</td><td>checkbox
<table>
<tr><td>1</td><td>ipv_where___1</td><td>head</td></tr>
<tr><td>2</td><td>ipv_where___2</td><td>mouth</td></tr>
<tr><td>3</td><td>ipv_where___3</td><td>throat</td></tr>
<tr><td>4</td><td>ipv_where___4</td><td>neck</td></tr>
<tr><td>5</td><td>ipv_where___5</td><td>back</td></tr>
<tr><td>6</td><td>ipv_where___6</td><td>chest</td></tr>
<tr><td>7</td><td>ipv_where___7</td><td>armpits</td></tr>
<tr><td>8</td><td>ipv_where___8</td><td>breasts</td></tr>
<tr><td>9</td><td>ipv_where___9</td><td>stomach/abdomen</td></tr>
<tr><td>10</td><td>ipv_where___10</td><td>my port or PICC line</td></tr>
<tr><td>11</td><td>ipv_where___11</td><td>my arms or legs</td></tr>
<tr><td>12</td><td>ipv_where___12</td><td>other</td></tr>
</table></td></tr>
</table>

<table>
<tr><td></td><td>36</td><td>[vest_dislike]

Show the field ONLY if:
[ever_used(3)] = '1'</td><td>What do you DISLIKE about High Frequency Chest Wall Oscillation (Vest)?
Select ALL that apply</td><td>checkbox<table>
<tr><td>1</td><td>vest_dislike___1</td><td>I get short of breath</td></tr>
<tr><td>2</td><td>vest_dislike___2</td><td>It takes too long</td></tr>
<tr><td>3</td><td>vest_dislike___3</td><td>It is painful</td></tr>
<tr><td>4</td><td>vest_dislike___4</td><td>It is uncomfortable</td></tr>
<tr><td>5</td><td>vest_dislike___5</td><td>It is less effective than other forms of airway clearance</td></tr>
<tr><td>6</td><td>vest_dislike___6</td><td>It does not work well with my other treatments</td></tr>
<tr><td>7</td><td>vest_dislike___7</td><td>It is unpleasant or embarrassing</td></tr>
<tr><td>8</td><td>vest_dislike___8</td><td>Nothing</td></tr>
<tr><td>9</td><td>vest_dislike___9</td><td>I don't know</td></tr>
</table></td></tr>
<tr><td></td><td>37</td><td>[vest_follow_up]

Show the field ONLY if:
[vest_dislike(3)] = '1' or [vest_dislike(4)] = '1' or [vest_dislike(7)] = '1'</td><td>Why is the High Frequency Chest Wall Oscillation (Vest) painful, uncomfortable or unpleasant?</td><td>text</td></tr>
<tr><td></td><td>38</td><td>[vest_where]

Show the field ONLY if:
[vest_dislike(3)] = '1' or [vest_dislike(4)] = '1' or [vest_dislike(7)] = '1'</td><td>Where on your body does High Frequency Chest Wall Oscillation (Vest) bother you?
Select ALL that apply</td><td>checkbox<table>
<tr><td>1</td><td>vest_where___1</td><td>head</td></tr>
<tr><td>2</td><td>vest_where___2</td><td>mouth</td></tr>
<tr><td>3</td><td>vest_where___3</td><td>throat</td></tr>
<tr><td>4</td><td>vest_where___4</td><td>neck</td></tr>
<tr><td>5</td><td>vest_where___5</td><td>back</td></tr>
<tr><td>6</td><td>vest_where___6</td><td>chest</td></tr>
<tr><td>7</td><td>vest_where___7</td><td>armpits</td></tr>
<tr><td>8</td><td>vest_where___8</td><td>breasts</td></tr>
<tr><td>9</td><td>vest_where___9</td><td>stomach/abdomen</td></tr>
<tr><td>10</td><td>vest_where___10</td><td>my port or PICC line</td></tr>
<tr><td>11</td><td>vest_where___11</td><td>my arms or legs</td></tr>
<tr><td>12</td><td>vest_where___12</td><td>other</td></tr>
</table></td></tr>
<tr><td></td><td>39</td><td>[pep_dislike]

Show the field ONLY if:
[ever_used(4)] = '1'</td><td>What do you DISLIKE about Oscillating Positive Expiratory Pressure (PEP), e.g. Aerobika or Acapella?
Select ALL that apply</td><td>checkbox<table>
<tr><td>1</td><td>pep_dislike___1</td><td>I get short of breath</td></tr>
<tr><td>2</td><td>pep_dislike___2</td><td>It takes too long</td></tr>
<tr><td>3</td><td>pep_dislike___3</td><td>It is painful</td></tr>
<tr><td>4</td><td>pep_dislike___4</td><td>It is uncomfortable</td></tr>
<tr><td>5</td><td>pep_dislike___5</td><td>It is less effective than other forms of airway clearance</td></tr>
<tr><td>6</td><td>pep_dislike___6</td><td>It does not work well with my other treatments</td></tr>
<tr><td>7</td><td>pep_dislike___7</td><td>It is unpleasant or embarrassing</td></tr>
<tr><td>8</td><td>pep_dislike___8</td><td>Nothing</td></tr>
<tr><td>9</td><td>pep_dislike___9</td><td>I don't know</td></tr>
</table></td></tr>
<tr><td></td><td>40</td><td>[pep_follow_up]

Show the field ONLY if:
[pep_dislike(3)] = '1' or [pep_dislike(4)] = '1' or [pep_dislike(7)] = '1'</td><td>Why is Oscillating Positive Expiratory Pressure (PEP), e.g. Aerobika or Acapella painful, uncomfortable or unpleasant?</td><td>text</td></tr>
</table>

	41	[pep_where]<br><br>Show the field ONLY if:<br>[pep_dislike(3)] = '1' or [pep_dislike(4)] = '1' or [pep_dislike(7)] = '1'	Where on your body does Oscillating Positive Expiratory Pressure (PEP), e.g. Aerobika or Acapella bother you?<br>Select ALL that apply	checkbox<br>1 pep_where___1 head<br>2 pep_where___2 mouth<br>3 pep_where___3 throat<br>4 pep_where___4 neck<br>5 pep_where___5 back<br>6 pep_where___6 chest<br>7 pep_where___7 armpits<br>8 pep_where___8 breasts<br>9 pep_where___9 stomach/abdomen<br>10 pep_where___10 my port or PICC line<br>11 pep_where___11 my arms or legs<br>12 pep_where___12 other
	42	[exercise_dislike]<br><br>Show the field ONLY if:<br>[ever_used(5)] = '1'	What do you DISLIKE about Vigorous Aerobic Exercise?<br>Select ALL that apply	checkbox<br>1 exercise_dislike___1 I get short of breath<br>2 exercise_dislike___2 It takes too long<br>3 exercise_dislike___3 It is painful<br>4 exercise_dislike___4 It is uncomfortable<br>5 exercise_dislike___5 It is less effective than other forms of airway clearance<br>6 exercise_dislike___6 It does not work well with my other treatments<br>7 exercise_dislike___7 It is unpleasant or embarrassing<br>8 exercise_dislike___8 Nothing<br>9 exercise_dislike___9 I don't know
	43	[exercise_follow_up]<br><br>Show the field ONLY if:<br>[exercise_dislike(3)] = '1' or [exercise_dislike(4)] = '1' or [exercise_dislike(7)] = '1'	Why is Vigorous Aerobic Exercise painful, uncomfortable or unpleasant?	text
	44	[exercise_where]<br><br>Show the field ONLY if:<br>[exercise_dislike(3)] = '1' or [exercise_dislike(4)] = '1' or [exercise_dislike(7)] = '1'	Where on your body does Vigorous Aerobic Exercise bother you?<br>Select ALL that apply	checkbox<br>1 exercise_where___1 head<br>2 exercise_where___2 mouth<br>3 exercise_where___3 throat<br>4 exercise_where___4 neck<br>5 exercise_where___5 back<br>6 exercise_where___6 chest<br>7 exercise_where___7 armpits<br>8 exercise_where___8 breasts<br>9 exercise_where___9 stomach/abdomen<br>10 exercise_where___10 my port or PICC line<br>11 exercise_where___11 my arms or legs<br>12 exercise_where___12 other

<table>
<tr><td></td><td>45</td><td>[huff_cough_dislike]

Show the field ONLY if:
[ever_used(6)] = '1'</td><td>What do you DISLIKE about Huff Coughing?
Select ALL that apply</td><td>checkbox<table>
<tr><td>1</td><td>huff_cough_dislike___1</td><td>I get short of breath</td></tr>
<tr><td>2</td><td>huff_cough_dislike___2</td><td>It takes too long</td></tr>
<tr><td>3</td><td>huff_cough_dislike___3</td><td>It is painful</td></tr>
<tr><td>4</td><td>huff_cough_dislike___4</td><td>It is uncomfortable</td></tr>
<tr><td>5</td><td>huff_cough_dislike___5</td><td>It is less effective than other forms of airway clearance</td></tr>
<tr><td>6</td><td>huff_cough_dislike___6</td><td>It does not work well with my other treatments</td></tr>
<tr><td>7</td><td>huff_cough_dislike___7</td><td>It is unpleasant or embarrassing</td></tr>
<tr><td>8</td><td>huff_cough_dislike___8</td><td>Nothing</td></tr>
<tr><td>9</td><td>huff_cough_dislike___9</td><td>I don't know</td></tr>
</table></td></tr>
<tr><td></td><td>46</td><td>[huff_cough_follow_up]

Show the field ONLY if:
[huff_cough_dislike(3)] = '1' or
[huff_cough_dislike(4)] = '1' or
[huff_cough_dislike(7)] = '1'</td><td>Why is Huff Coughing painful, uncomfortable or unpleasant?</td><td>text</td></tr>
<tr><td></td><td>47</td><td>[huff_cough_where]

Show the field ONLY if:
[huff_cough_dislike(3)] = '1' or
[huff_cough_dislike(4)] = '1' or
[huff_cough_dislike(7)] = '1'</td><td>Where on your body does Huff Coughing bother you?
Select ALL that apply</td><td>checkbox<table>
<tr><td>1</td><td>huff_cough_where___1</td><td>head</td></tr>
<tr><td>2</td><td>huff_cough_where___2</td><td>mouth</td></tr>
<tr><td>3</td><td>huff_cough_where___3</td><td>throat</td></tr>
<tr><td>4</td><td>huff_cough_where___4</td><td>neck</td></tr>
<tr><td>5</td><td>huff_cough_where___5</td><td>back</td></tr>
<tr><td>6</td><td>huff_cough_where___6</td><td>chest</td></tr>
<tr><td>7</td><td>huff_cough_where___7</td><td>armpits</td></tr>
<tr><td>8</td><td>huff_cough_where___8</td><td>breasts</td></tr>
<tr><td>9</td><td>huff_cough_where___9</td><td>stomach/abdomen</td></tr>
<tr><td>10</td><td>huff_cough_where___10</td><td>my port or PICC line</td></tr>
<tr><td>11</td><td>huff_cough_where___11</td><td>my arms or legs</td></tr>
<tr><td>12</td><td>huff_cough_where___12</td><td>other</td></tr>
</table></td></tr>
<tr><td></td><td>48</td><td>[treatments_maintain]</td><td>Section Header: My CF Treatments
Which treatments do you currently use to maintain your health?
Select ALL that apply</td><td>checkbox<table>
<tr><td>1</td><td>treatments_maintain___1</td><td>Inhaled Antibiotics: e.g. Tobramycin (TOBI), Aztreonam (Cayston), etc.</td></tr>
<tr><td>2</td><td>treatments_maintain___2</td><td>Oral Antibiotics: e.g. Azithromycin, Augmentin, Bactrim, Cephalexin, Ciprofloxacin, etc.</td></tr>
<tr><td>3</td><td>treatments_maintain___3</td><td>Mucus thinners: e.g. Hypertonic Saline, Dornase Alfa (Pulmozyme), etc.</td></tr>
<tr><td>4</td><td>treatments_maintain___4</td><td>None of these</td></tr>
</table></td></tr>
<tr><td></td><td>49</td><td>[pt_effects]</td><td>How does airway clearance affect your health?</td><td>radio<table>
<tr><td>1</td><td>It improves it</td></tr>
<tr><td>2</td><td>It does not change / no effect</td></tr>
<tr><td>3</td><td>It worsens it</td></tr>
</table></td></tr>
</table>

	50	[theratype]	Do you currently take any of these medications?	radio<br>1 ivacaftor (kalydeco)<br>2 lumacaftor/ivacaftor (orkambi)<br>3 tezacaftor/ivacaftor (symdeko)<br>4 elexacaftor/tezacaftor/ivacaftor (trikafta)<br>5 None of these
	51	[clearance_perform1]<br><br>Show the field ONLY if:<br>[theratype] = '1'	Which statement best describes your current airway clearance use since starting ivacaftor (kalydeco)?	radio<br>1 I perform more airway clearance now<br>2 I perform the same amount of airway clearance now<br>3 I perform less airway clearance now
	52	[clearance_perform2]<br><br>Show the field ONLY if:<br>[theratype] = '2'	Which statement best describes your current airway clearance use since starting lumacaftor/ivacaftor (orkambi)?	radio<br>1 I perform more airway clearance now<br>2 I perform the same amount of airway clearance now<br>3 I perform less airway clearance now
	53	[clearance_perform3]<br><br>Show the field ONLY if:<br>[theratype] = '3'	Which statement best describes your current airway clearance use since starting tezacaftor/ivacaftor (symdeko)?	radio<br>1 I perform more airway clearance now<br>2 I perform the same amount of airway clearance now<br>3 I perform less airway clearance now
	54	[clearance_perform4]<br><br>Show the field ONLY if:<br>[theratype] = '4'	Which statement best describes your current airway clearance use since starting elexacaftor/tezacaftor/ivacaftor (trikafta)?	radio<br>1 I perform more airway clearance now<br>2 I perform the same amount of airway clearance now<br>3 I perform less airway clearance now
	55	[mod_clearance_more]<br><br>Show the field ONLY if:<br>[clearance_perform1] = '1' or [clearance_perform2] = '1' or [clearance_perform3] = '1' or [clearance_perform4] = '1'	Why do you perform more airway clearance now?	notes
	56	[mod_clearance_same]<br><br>Show the field ONLY if:<br>[clearance_perform1] = '2' or [clearance_perform2] = '2' or [clearance_perform3] = '2' or [clearance_perform4] = '2'	Why do you perform the same amount of airway clearance now as you did prior to starting your modulator?	notes
	57	[mod_clearance_less]<br><br>Show the field ONLY if:<br>[clearance_perform1] = '3' or [clearance_perform2] = '3' or [clearance_perform3] = '3' or [clearance_perform4] = '3'	Why do you perform less airway clearance now?	notes
	58	[ranking_modulators]<br><br>Show the field ONLY if:<br>[theratype] = '1' or [theratyp e] = '2' or [theratype] = '3' or [theratype] = '4'	Section Header: *Please indicate how important each of the following are for maintaining your health:*<br><br>CFTR Modulators<br>Kalydeco, Orkambi, Symdeko or Trikafta	slider<br>Slider labels: Least Important, , Most Important<br>Custom alignment: RH
	59	[ranking_inhaled_anti]<br><br>Show the field ONLY if:<br>[treatments_maintain(1)] = '1'	Inhaled Antibiotics<br>e.g. Tobramycin (TOBI), Aztreonam (Cayston), etc.	slider<br>Slider labels: Least Important, , Most Important<br>Custom alignment: RH
	60	[ranking_oral_anti]<br><br>Show the field ONLY if:<br>[treatments_maintain(2)] = '1'	Oral Antibiotics<br>e.g. Azithromycin, Augmentin, Bactrim, Cephalexin, Ciprofloxacin, etc.	slider<br>Slider labels: Least Important, , Most Important<br>Custom alignment: RH

	61	[ranking_mucus]<br><br>Show the field ONLY if:<br>[treatments_maintain(3)] = '1'	Mucus thinners<br>e.g. Hypertonic Saline or Dornase Alfa (Pulmozyme), etc.	slider<br>Slider labels: Least Important, , Most Important<br>Custom alignment: RH
	62	[ranking_airway]	Airway clearance techniques<br>e.g. Manual Chest PT, IPV, Vest, or PEP (aerobika or acapella)	slider<br>Slider labels: Least Important, , Most Important<br>Custom alignment: RH
	63	[ranking_exercise]	Exercise	slider<br>Slider labels: Least Important, , Most Important<br>Custom alignment: RH
	64	[ranking_nutrition]	Nutrition	slider<br>Slider labels: Least Important, , Most Important<br>Custom alignment: RH
	65	[race]<br><br>Show the field ONLY if:<br>[who_filled_out] = '1' or [who_filled_out] = '2'	Section Header: *Demographics*<br><br>What is your race?<br>Select ALL that apply	checkbox<br>1 | race___1 | American Indian or Alaska Native<br>2 | race___2 | Asian<br>3 | race___3 | Black or African American<br>4 | race___4 | Hispanic or Latino<br>5 | race___5 | Native Hawaiian or Other Pacific Islander<br>6 | race___6 | White<br>7 | race___7 | Other (not represented above)<br>8 | race___8 | Prefer not to answer
	66	[race2]<br><br>Show the field ONLY if:<br>[who_filled_out] = '3'	What is THE PATIENT'S race?<br>Select ALL that apply	checkbox<br>1 | race2___1 | American Indian or Alaska Native<br>2 | race2___2 | Asian<br>3 | race2___3 | Black or African American<br>4 | race2___4 | Hispanic or Latino<br>5 | race2___5 | Native Hawaiian or Other Pacific Islander<br>6 | race2___6 | White<br>7 | race2___7 | Other (not represented above)<br>8 | race2___8 | Prefer not to answer
	67	[patient_household_size]	How many people are currently living in your household, including yourself?	text
	68	[patient_household_children]	How many children ≤18 years old live in your house, including yourself?	text
	69	[patient_education]<br><br>Show the field ONLY if:<br>[who_filled_out] = '1' or [who_filled_out] = '2'	What is the highest grade you have completed?	radio<br>1 | Preschool or Younger<br>2 | Elementary (Kindergarten - 5th Grade)<br>3 | Middle School (6th - 8th Grade)<br>4 | High School: (9th - 12th grade)<br>5 | College/Junior College<br>6 | Graduate School or Beyond
	70	[patient_education_2]<br><br>Show the field ONLY if:<br>[who_filled_out] = '3'	What is the highest grade your child (THE PATIENT) has completed?	radio<br>1 | Preschool or Younger<br>2 | Elementary (Kindergarten - 5th Grade)<br>3 | Middle School (6th - 8th Grade)<br>4 | High School: (9th - 12th grade)<br>5 | College/Junior College<br>6 | Graduate School or Beyond

	71	[parent_degree_2]<br><br>Show the field ONLY if:<br>[who_filled_out] = '1'	What is the highest degree your parent or caregiver has earned?	radio<br>1 Some high school or less<br>2 High school diploma or equivalency (GED)<br>3 Associate degree (junior college) or vocational degree/license<br>4 Bachelor's degree<br>5 Master's degree<br>6 Doctorate, Professional (MD, JD, DDS, PhD, etc)<br>7 None of the above
	72	[parent_degree]<br><br>Show the field ONLY if:<br>[who_filled_out] = '3'	What is the highest degree you (THE PARENT/CAREGIVER) earned?	radio<br>1 Some high school or less<br>2 High school diploma or equivalency (GED)<br>3 Associate degree (junior college) or vocational degree/license<br>4 Bachelor's degree<br>5 Master's degree<br>6 Doctorate, Professional (MD, JD, DDS, PhD, etc)<br>7 None of the above
	73	[work_status]<br><br>Show the field ONLY if:<br>[who_filled_out] = '1' or [who_filled_out] = '2'	What is your current work or school status?	radio<br>1 Attending school outside the home<br>2 Attending classes from home<br>3 Working full or part time (either outside the home or at a home-based business)<br>4 Not attending school or working due to my health<br>5 Not working for other reasons<br>6 Too young for school, attending day care outside of home<br>7 Too young for school, attending day care within the home
	74	[work_status_2]<br><br>Show the field ONLY if:<br>[who_filled_out] = '3'	What is your child's (THE PATIENT'S) current work or school status?	radio<br>1 Attending school outside the home<br>2 Attending classes from home<br>3 Working full or part time (either outside the home or at a home-based business)<br>4 Not attending school or working due to my health<br>5 Not working for other reasons<br>6 Too young for school, attending day care outside of home<br>7 Too young for school, attending day care within the home
	75	[who_completed]	Who completed this survey?<br>Select ALL that apply	checkbox<br>1 who_completed___1 Patient<br>2 who_completed___2 Patient's Parent / Caregiver<br>3 who_completed___3 Patient's Spouse<br>4 who_completed___4 Other
	76	[pt_survey_complete]	Section Header: *Form Status*<br><br>Complete?	dropdown<br>0 Incomplete<br>1 Unverified<br>2 Complete
